# DIS3 isoforms vary in their endoribonuclease activity and are differentially expressed within haematological cancers

**DOI:** 10.1042/BCJ20170962

**Published:** 2018-06-29

**Authors:** Sophie R. Robinson, Sandra C. Viegas, Rute G. Matos, Susana Domingues, Marisa Bedir, Helen J.S. Stewart, Timothy J. Chevassut, Antony W. Oliver, Cecilia M. Arraiano, Sarah F. Newbury

**Affiliations:** 1 Medical Research Building, Brighton and Sussex Medical School, University of Sussex, Falmer, Brighton BN1 9PS, U.K.; 2 Instituto de Tecnologia Química e Biológica António Xavier, Universidade Nova de Lisboa, Av. da República, 2780-157 Oeiras, Portugal; 3 School of Life Sciences, Genome Damage and Stability Centre, University of Sussex, Falmer, Brighton BN1 9RQ, U.K.

**Keywords:** CMML, DIS3, myeloma, PIN domain, RNA stability

## Abstract

DIS3 (defective in sister chromatid joining) is the catalytic subunit of the exosome, a protein complex involved in the 3′–5′ degradation of RNAs. DIS3 is a highly conserved exoribonuclease, also known as Rrp44. Global sequencing studies have identified DIS3 as being mutated in a range of cancers, with a considerable incidence in multiple myeloma. In this work, we have identified two protein-coding isoforms of DIS3. Both isoforms are functionally relevant and result from alternative splicing. They differ from each other in the size of their N-terminal PIN (PilT N-terminal) domain, which has been shown to have endoribonuclease activity and tether DIS3 to the exosome. Isoform 1 encodes a full-length PIN domain, whereas the PIN domain of isoform 2 is shorter and is missing a segment with conserved amino acids. We have carried out biochemical activity assays on both isoforms of full-length DIS3 and the isolated PIN domains. We find that isoform 2, despite missing part of the PIN domain, has greater endonuclease activity compared with isoform 1. Examination of the available structural information allows us to provide a hypothesis to explain this altered behaviour. Our results also show that multiple myeloma patient cells and all cancer cell lines tested have higher levels of isoform 1 compared with isoform 2, whereas acute myeloid leukaemia and chronic myelomonocytic leukaemia patient cells and samples from healthy donors have similar levels of isoforms 1 and 2. Taken together, our data indicate that significant changes in the ratios of the two isoforms could be symptomatic of haematological cancers.

## Introduction

DIS3 (defective in sister chromatid joining) is a highly conserved RNA exoribonuclease and a catalytic subunit of the exosome, a protein complex involved in the 3′–5′ degradation and processing of RNAs. The crucial role that DIS3 plays within RNA processing and decay is highlighted by its association with many forms of human cancer [1,2]. High-throughput studies have identified that DIS3 is recurrently mutated in different types of cancer such as multiple myeloma (10%) and in acute myeloid leukaemia (AML, 4%) [3–7]. Additionally, DIS3 is differentially expressed in superficial spreading melanoma and nodular melanoma and has also been identified as a candidate oncogene in colorectal cancer [8–11]. Therefore, the levels of DIS3 protein appear to be important and relevant to the progression of many commonly occurring cancers.

The precise role of DIS3 in cancer progression is not at all clear. Indeed, knockdown or mutation of DIS3 in model organisms or human tissue culture cells results in lethality or inhibition of proliferation. For example, in both the yeast *Saccharomyces cerevisiae* and in *Drosophila*, knockdown of DIS3 has been shown to result in lethality, due to failure in mitosis [12,13]. Similarly, in human HEK293 cells expressing catalytically dead mutants in DIS3, where the endogenous activity of DIS3 has been knocked down, the cells proliferate more slowly and have reduced metabolic activity [14]. DIS3 is found in the nucleus of human cells and has been shown to be involved in the degradation of a vast range of RNAs including protein-coding RNAs, snoRNA precursors, introns, long non-coding RNAs, microRNAs and tRNAs as well as Promoter Upstream Transcripts (PROMPTs) and Cryptic Unstable Transcripts (CUTs) [15]. Therefore, inhibition of its activity is likely to have wide-ranging effects on cellular metabolism.

**Figure 1.**
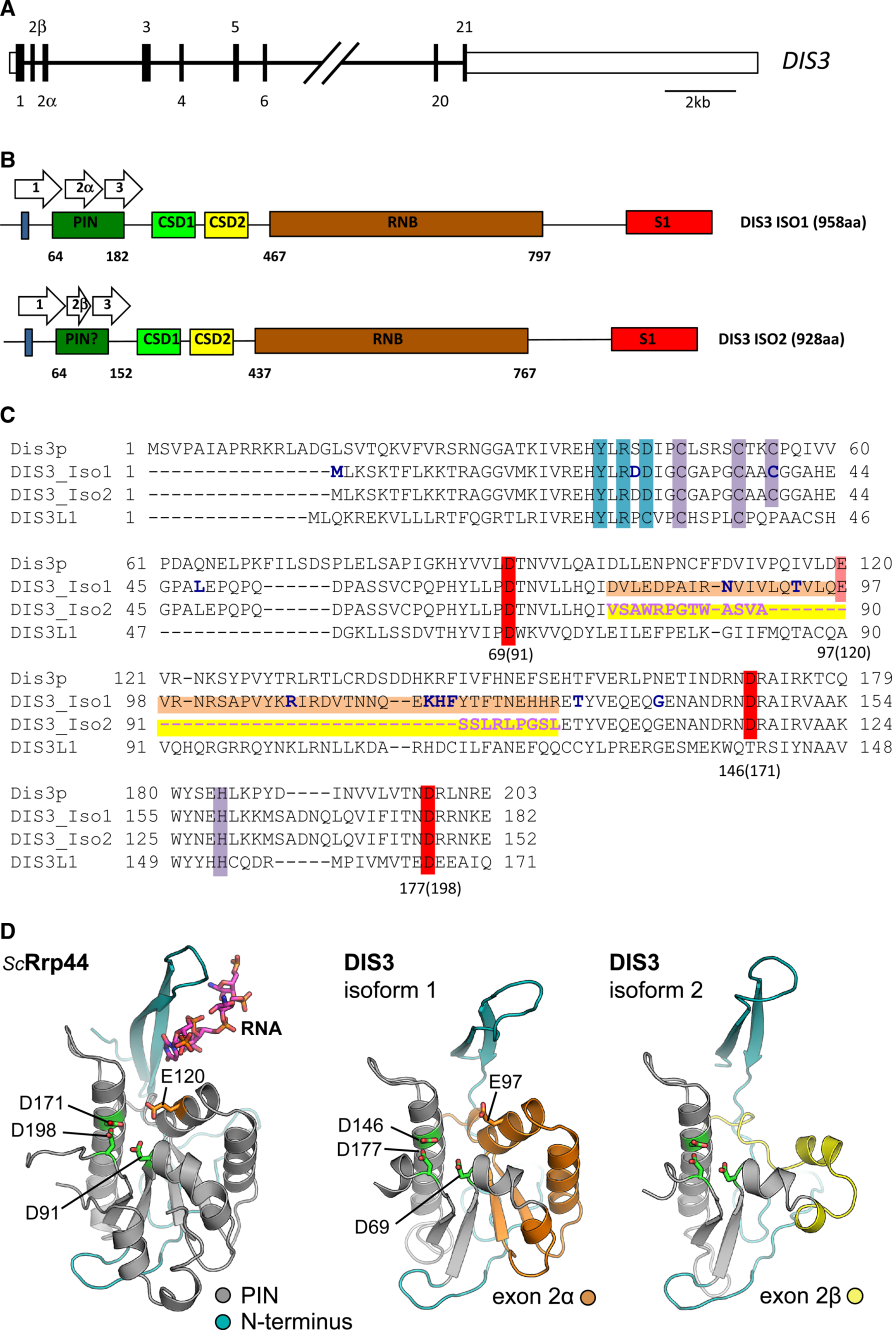
Schematic of the DIS3 locus together with the sequence and structure of the N-terminus of human DIS3. (**A**) The DIS3 gene encodes two alternate exons, exon 2α and 2β that are incorporated into isoforms 1 and 2 transcripts, respectively. (**B**) Exon 2 encodes a large portion of the PIN domain. Exon 2β is smaller than exon 2α, resulting in a PIN domain 30 amino acids shorter in the isoform 2 protein. Although the rest of the protein remains in frame, this results in isoform 2 being 30 amino acids shorter in total than isoform 1. (**C**) Diagram showing the amino acid residues corresponding to the N-terminus and PIN domain of *S. cerevisiae* Dis3p, human DIS3 isoform 1, human DIS3 isoform 2, and human DIS3L1, together with residues thought to be important in their activity. The amino acid residues corresponding to isoform 1 are highlighted in orange, whereas the residues corresponding to isoform 2 are highlighted in yellow. The residues important for catalysis are highlighted in red with numbers below referring to human Dis3 isoform 1 amino acid sequence with corresponding yeast Dis3p sequence numbers in brackets. Acidic residues which are not important for catalysis in the SMG6 PIN domain are represented in pale red. The N-terminal Y-R-D exosome-binding site is marked in blue and the conserved CCCH motif is marked in purple [20]. Residues that are frequently mutated in multiple myeloma are marked in bold blue letters. (**D**) Comparisons of the *S. cerevisiae* PIN domain with the human isoforms 1 and 2. In isoform 2, the loss of two alpha helices (labelled orange in isoform 1) is predicted to be highly destabilizing and affect the activity of this isoform. The three aspartic acid residues known to be important in co-ordinating the metal ion required for cleavage activity (D69, 146, and 177 in Dis3^iso1^) are marked in green. Co-ordinates corresponding to the PIN domain of ScRrp44 were extracted from PDB entry 4IFD [26]. Sequence-threaded homology models for both isoforms of human DIS3 were generated using the Phyre2 web-server [33].

Human DIS3 is a multi-domain protein containing two different catalytic activities: a 3′–5′ exonucleolytic activity conferred by the RNaseII/R (RNB) domain [16] and an endonucleolytic activity via the PilT N-terminal (PIN) domain [17–19]. Other domains include a CR3 motif [20], two cold shock domains (CSDs) and an S1 domain which non-specifically binds RNA [21] (Figure 1). The human genome contains two further homologues: DIS3L1 (DIS3L), which contains an inactive PIN domain, and DIS3L2, where the PIN domain is absent [22–25]. In mammals, DIS3 functions as one of the three catalytic subunits of the exosome, along with DIS3L1 and Rrp6, a distributive exoribonuclease which belongs to the RNase D family [26,27]. The RNA substrate is usually guided through the central channel within the 9-subunit exosome barrel and then degraded by the exonuclease domain of DIS3 located at the base of the ring [26,28–30]. Alternatively, some substrates, such as highly structured RNA molecules, may access the DIS3 catalytic centre by direct entry rather than threading through the exosome barrel [28,29,31].

The importance of the PIN domain for DIS3 activity is demonstrated by evidence, showing that mutations which abrogate the endonuclease activity of the PIN domain together with mutations in the exonuclease domain have a synergistic effect on proliferation and metabolic activity in both human and yeast cells [14,18,19]. The PIN domain is a compact domain of ∼100 amino acids which is present in many diverse proteins from all kingdoms of life. It is commonly found in RNA processing enzymes; for example, those involved in non-sense-mediated decay, such as SMG5 and SMG6 [32]. PIN domains contain three to four conserved acidic amino acid residues which serve to co-ordinate a divalent metal ion catalyzing cleavage of the RNA and release of nucleotide 5′ monophosphate products [17–19]. Crystal structure studies on the *S. cerevisiae* exosome bound to Rrp44 show that residues within the PIN domain contact the RNA together with regions of Rrp41 and Rrp43 (core exosome proteins) [26]. Furthermore, the N-terminal CR3 motif together with the YxRxD motif at the N-terminal of the PIN domain provides further anchoring of Rrp44 to the exosome barrel [20]. Taken together, these interactions produce a channel for RNA binding, directing it to the catalytic centres.

In the present paper, we identify, for the first time, the presence of two alternatively spliced protein-coding isoforms of human DIS3 that differ in the region encoding the crucial PIN domain. The shorter isoform (isoform 2) is missing conserved amino acid residues in the centre of the PIN domain. Biochemical assays using purified full-length DIS3 isoforms, as well as isolated PIN domains, demonstrate that isoform 1 has reduced endonuclease activity compared with isoform 2. Examination of the available structural information allows us to provide a hypothesis to explain this altered behaviour. *In vivo*, both of the isoforms are ubiquitously expressed but display differential expression levels in primary patient cells and many cell lines. Our results indicate that multiple myeloma patient cells and all cancer cell lines tested have higher levels of isoform 1 compared with isoform 2, whereas AML and chronic myelomonocytic leukaemia (CMML) patient samples and samples from healthy donors have similar levels of isoforms 1 and 2. Taken together, our data indicate that aberrant expression of these two isoforms can contribute to the progression of haematological cancers.

## Methods

### Overexpression, purification and *in vitro* activity assays of human Dis3 isoforms

The pReceiver-B03 vectors containing recombinant DIS3 isoform 1 or 2 were purchased from GeneCopoeia. This vector contains a T7 promoter for high-level expression, GST-tag and an ampicillin resistance gene for bacterial selection. The presence of each isoform was confirmed by DNA sequencing. To construct the truncated versions of DIS3 isoforms (ISO1 and ISO2), the expression plasmid (vector H0869 and vector H3667, respectively) was amplified by PCR with primers that cover the entire vector sequence, but create a premature termination codon after the amino acid N219 (N189 on ISO2) downstream from the PIN domain. In this way, the vectors created (pRSV-1 and pRSV-2) enable the expression of a truncated version of each protein composed only by the respective PIN domain fused to the protein purification tag. The PCR products were circularized with T4 DNA ligase and used to transform competent DH5α strains. The sequence of the selected clones was confirmed by DNA sequencing.

DIS3 protein isoforms ISO1 and ISO2, and the truncated versions containing only the respective PIN domain were overproduced with a glutathione *S*-transferase (GST) tag in *Escherichia coli* BL21-CodonPlus(DE3)-RIL strain containing the recombinant plasmids of interest. As a control, we have overexpressed the GST protein alone in the same strain containing the commercial plasmid pGEX-4T1 (GE Healthcare). The five proteins were purified by affinity chromatography. Briefly, cells were grown at 30°C in LB medium supplemented with ampicillin and chloramphenicol to an optical density (600 nm) near 1. Protein expression was induced by the addition of 0.5 mM of IPTG for 8 h at 20°C and cells were harvested by centrifugation. The culture pellets were resuspended in 1/20 volumes of Buffer A [20 mM Tris–HCl (pH 7.5), 150 mM NaCl, and 2 mM DTT]. Suspensions were lysed using a French Press at 1000 Psi in the presence of protease inhibitors. After lysis, the crude extracts were treated with 125 U of Benzonase (Sigma) to degrade nucleic acids and clarified by a 60 min centrifugation at 18 000 rpm, 4°C. The GST-tagged recombinant proteins were purified by affinity chromatography, using the ÄKTA FPLC™ System (GE Healthcare). The clarified extracts were loaded onto a GST-Trap 1 ml column (GE Healthcare) previously equilibrated in Buffer A. Protein elution was achieved with Buffer A with 20 mM reduced glutathione. The fractions containing the protein of interest, free of contaminants, were pooled and the buffer was exchanged for 20 mM Tris–HCl (pH 8), 100 mM KCl and 50% glycerol using Sephadex G-25 PD-10 desalting columns (GE Healthcare). Proteins were quantified using the Bradford Method and stored at −20°C. The purity of the enzymes was analyzed by sodium dodecyl sulfate–polyacrylamide gel electrophoresis (SDS–PAGE) and by western blot using anti-GST antibodies, revealing >90% homogeneity.

*In vitro* activity assays of the proteins were performed using the synthetic 30-mer oligoribonucleotide ss16-A_14_ as a substrate, labelled at its 5′-end with [γ-^32^ATP] and T4 Polynucleotide Kinase (Ambion), and circularized with T4 RNA ligase (Thermo). Protein and RNA concentrations were 50 and 25 nM, respectively, in all the cases. The experiment was performed in a buffer containing 20 mM HEPES (pH 7.5), 150 mM NaCl, 3 mM MnCl_2_, and 1 mM DTT.

The reactions, in a total volume of 40 µl, were started by the addition of the enzyme and further incubated at 37°C. Aliquots of 5 µl were withdrawn at different time points, and the reactions were stopped by the addition of formamide containing dye supplemented with 10 mM EDTA. As a control, an aliquot of each reaction, without the enzyme, was incubated until the end of the assay. Reaction products were resolved in a 7 M urea/20% polyacrylamide gel and visualized by phosphorImaging (FLA-2000, Fuji, Stamford, CT, U.S.A.). Each activity assay was performed at least in triplicate. Quantification of the disappearance of the substrate was performed using the ImageQuant software (GE Healthcare).

For methods relating to Supplementary Figure S5, the reader is referred to the Supplementary Methods section. Plasmids and primers used in the present study are listed in Supplementary Tables S1–S3.

### Structural analyses

Sequence-threaded homology models of human DIS3 were generated using the structure of *S. cerevisiae* Rrp44, in the context of an 11-subunit exosome complex (PDB: 5K36), as a template [33].

### Cell culture

Cells were cultured in Dulbecco's modified Eagle's medium (HeLa, HEK-293), RPMI-1640 medium (OCI-AML3, U-266, RPMI-8226, THP-1, DG-75, GM12878, KG-1, KMS-12-BM, MOLP-8) or DMEM-F12 (U-2OS, SAOS-2) with 10% foetal calf serum (FCS), supplemented with 2 mM l-glutamine (Gibco) and antibiotics (100 IU/ml penicillin and 100 µg/ml streptomycin, Sigma–Aldrich), at 37°C in a 5% CO_2_ humidified atmosphere. A total of 13 human cell lines were used in this work: the sources of these cell lines are given in Supplementary Table S4.

All patient samples were primary bone marrow aspirates with the exception of the healthy individuals which were peripheral blood samples. Primary bone marrow aspirates and peripheral blood samples were taken from routine diagnostic specimens after informed consent of the patients. The project received approval from the local ethics committee (The Brighton Blood Disorder Study, references: 09/025/CHE and 09/H1107/1) and was conducted in accordance with the Declaration of Helsinki. Twelve of the CMML samples were obtained from Cambridge Blood and Stem Cell Biobank. Further information on patient samples is given in Supplementary Table S5. Mononuclear cells were isolated by Histopaque 1077 density gradient purification as per the manufacturer's instructions.

### Polymerase chain reaction and sequencing

Genomic DNA was extracted using the DNeasy Kit (Qiagen) according to the manufacturer's instructions. Semi-quantitative RT-PCR was used to investigate the presence of alternatively spliced isoforms of DIS3 as well as for amplification of cDNA from each isoform for sequencing. PCR products were purified using the DNA purification kit (Qiagen) according to manufacturer's instructions, before being sent to Eurofins MWG for sequencing.

### RNA extraction and qRT-PCR

Cells (2 × 10^6^) were lysed and total RNA was extracted using the RNAeasy Mini Kit (Qiagen) with an on-column DNase digestion, as per the manufacturer's instructions. RNA concentrations were measured on a NanoDrop 1000 spectrophotometer (Thermo Scientific). RNA was reverse-transcribed using the High-Capacity cDNA Reverse Transcription Kit (Applied Biosystems) as per the manufacturer's instructions. qRT-PCR was performed on each cDNA sample in triplicate using TaqMan Universal PCR Master Mix, No AmpErase UNG (Life Technologies). Each of the DIS3 isoform 1- or isoform 2-specific custom-designed Taqman assays (Supplementary Table S3) (Life Technologies) were run on a ViiA7 qRT-PCR machine (Applied Biosystems). Negative controls lacking reverse transcriptase showed negligible background. All data were normalized to GAPDH and relative expression levels were calculated using the $2^{-\Delta \Delta C_{t}}$ method.

The amplification efficiencies of the DIS3 isoform-specific primers (DIS31 and DIS32) were determined using a series of cDNA dilutions (6.25, 12.5, 25, and 50 ng per 10 µl qPCR, based on RNA concentrations) using a standard curve set-up (Supplementary Figure S1).

### Statistical analysis

Unless otherwise stated, error bars represent the SEM obtained from three or more independent experiments. All statistics were carried out using the GraphPad PRISM software (V.6.01) and *, **, *** represent statistical significance at the levels of *P* < 0.05, *P* < 0.01 and *P* < 0.001, respectively.

## Results

### Characterization of two DIS3 protein-coding isoforms

Examination of the DIS3 gene in the Genome Browser ENSEMBL reveals five different DIS3 transcript annotations. Of the three transcripts listed as protein coding, two are annotated by the Consensus CDS project (CCDS) indicating consistent, high-quality annotation across the different annotation platforms. These two transcripts appear to differ in the use of a mutually exclusive exon 2, which encodes a large region of the endonucleolytic PIN domain and the length of their 3′-UTR (untranslated region). The PIN domain provides DIS3 with endonucleolytic activity and is thought to function in releasing natural exosome substrates that are stalled at sites of strong secondary structure [2,17].

Figure 1 shows the structure of the DIS3 gene. In humans, the length of the two annotated protein coding transcripts are 10 604 and 5232 nt, which are predicted to encode proteins of 958 and 928 amino acids (109 and 105 kDa, respectively) (www.genecards.org). Notably, exon 2α (DIS3 isoform 1) is longer than exon 2β (DIS3 isoform 2). If isoform 2 is translated, the inclusion of exon 2β would result in a PIN domain 30 amino acids shorter than isoform 1 while leaving the rest of the protein in frame (Figure 1C). As the total length of the PIN domain in isoform 1 is 118 amino acids, translation of isoform 2 would reduce its size by over 25%.

Sequence-threaded homology models, using the published structure of *S. cerevisiae* Rrp44 as a template [26,33], enabled us to make predictions about the effect of exon 2β on the structure and function of the human DIS3 PIN domain. Amino acids 78–129 (51 amino acids; encoded by exon 2α) form approximately half of the PIN domain fold (coloured orange in Figure 1D). This is replaced by the much shorter sequence (22 amino acids) encoded by exon 2β (yellow, Figure 1D). The loss of two β-strands, which serve to create the central β-sheet of the PIN domain, is likely to be destabilizing, at least in the context of the PIN domain alone. Interestingly, three highly conserved aspartic acid residues (D69, D177, and D146) essential for PIN endonuclease activity are present in a similar conformation in both isoforms (Figure 1D). These data therefore suggest that the DIS3 protein encoded by isoform 2 may have a different biochemical activity than that encoded by isoform 1 because of the altered size of the PIN domain.

To obtain an indication of whether both DIS3 isoform transcripts are translated into protein and thus functionally relevant, the online tool GWIPS (Genome Wide Information on Protein Synthesis) was used to analyze and visualize Ribo-seq data obtained using the ribosome profiling technique. Data from all ribosome profiling studies indicate that both isoforms are translated and although coverage is much lower on the shorter exon 2 of isoform 2 (exon 2β), ribosome binding is above the background level (Supplementary Figure S2). Therefore, isoform 2 can be translated into protein, at least under certain cellular conditions.

### Biochemical analyses of the endonuclease activities of Dis3 isoforms

**Figure 2.**
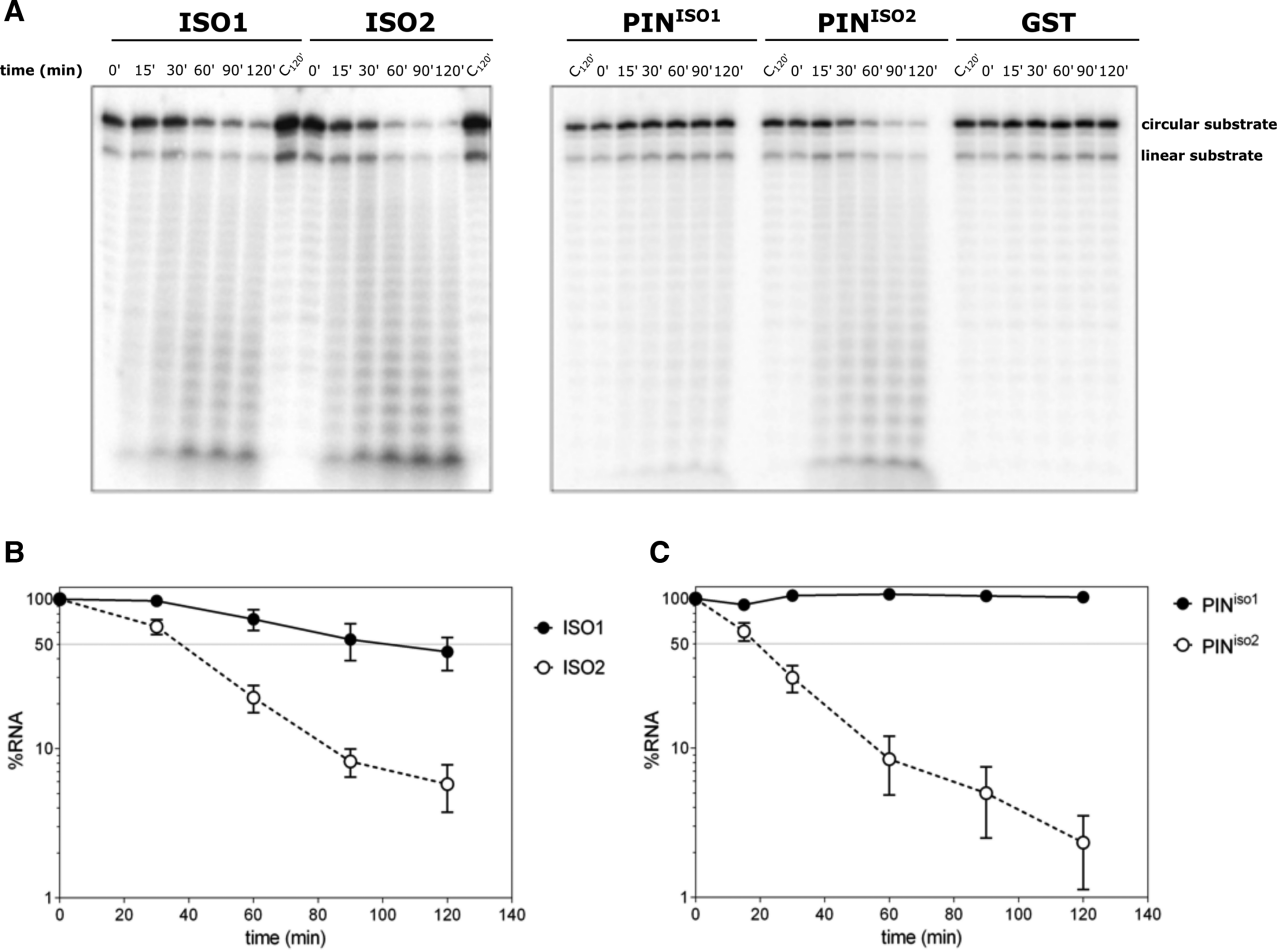
The shorter human DIS3 isoform 2 exhibits more endoribonuclease activity than isoform 1 on circular RNA substrates. (**A**) *In vitro* reactivity of purified wild-type full-length human DIS3 (ISO1) and the shorter variant ISO2 on a 5′-labelled 30-mer ssRNA circularized oligonucleotide. The same circularized substrate was incubated with a purified truncated version of human DIS3 isoforms 1 and 2 containing only the PIN domain (PIN^iso1^ and PIN^iso2^, respectively). Incubation times are indicated on top of the panels. In each case, the shorter variant isoform 2 (ISO2 and PIN^iso2^) shows a higher level of cleavage of the circular substrate than the longer isoform 1 (ISO1 and PIN^iso1^). The control GST-tag protein (empty vector purification containing only the GST-tag) shows no endoribonuclease cleavage (right-hand panel), showing that no contaminating ribonuclease activity is carried over during the purification process. (**B** and **C**) Graphical representation of the *in vitro* endoribonuclease activity of the full-length DIS3 isoform 1 (ISO1) compared with DIS3 isoform 2 (ISO2) (**B**) and the isoform 1 PIN domain (PIN^iso1^) compared with the isoform 2 PIN domain (PIN^iso2^) (**C**). The substrate consumption was quantified over time and represented as the percentage of RNA disappearance.

Truncation of the PIN domain in DIS3 isoform 2 would suggest altered or absent endoribonuclease activity. To test this, we expressed and purified both isoforms of full-length DIS3 (DIS3^iso1^ (ISO1) and DIS3^iso2^(ISO2)) as well as the isolated PIN domains (PIN^iso1^ and PIN^iso2^). All four DIS3 variants were assayed for endoribonuclease activity using a circularized ss16-A_14_ RNA substrate. Reactions were carried out in a high manganese concentration buffer, which favours DIS3 endonucleolytic activity [17–19]. If endoribonuclease activity occurs, the levels of the circular RNA substrate would be expected to decrease due to endonucleolytic cleavage. As can be seen from Figure 2, under our experimental conditions, ISO1, ISO2, and PIN^iso2^ have endoribonuclease activity, as the levels of circular RNA substrate decrease over time. Surprisingly, ISO2, with the truncated PIN domain, had a higher endoribonuclease activity than ISO1. A graphical comparison of their activity shows that 50% of the circular substrate is cleaved in 40 min for ISO2, whereas ISO1 is only capable of cleaving 10% of the substrate in the same time (Figure 2). In confirmation of this result, the shorter PIN domain PIN^iso2^ also cleaved the circular substrate more rapidly than PIN^iso1^ with 50% being cleaved in 20 min for PIN^iso2^, whereas PIN^iso1^ shows highly impaired activity. Concomitantly, there is also a higher accumulation of reaction products for PIN^iso2^ compared with PIN^iso1^. Therefore, truncation of the PIN domain in isoform 2 actually appears to result in a greater endoribonuclease activity, rather than the expected reduction. No degradation of substrate was seen for a GST-only control, indicating no evidence of contamination by bacterial ribonucleases (Figure 2A).

In PIN domains, three to four conserved acidic amino acid residues co-ordinate the metal ion in the active site [32,34,35] (D69, E97, D146, and D177 in the human DIS3 protein) [17–19]. The third of these residues, an aspartic acid (D146 in hDIS3), is the most conserved in the PIN domains (except in the PIN domain of DIS3L1 homologue) and its single mutation is reported to abolish its activity *in vivo* and *in vitro* [19,32,34,35]. The second one (E97 in hDIS3) is not strictly conserved across the PIN-domain family [32]. The truncated PIN domain of hDIS3 isoform 2, however, contains only three of these acidic amino acids (D69, D146, and D177) that are conserved in identity and spatial arrangement with the PIN domains of yeast Rrp44, human SMG6, and isoform 1 of human DIS3 (Figure 1 and Supplementary Figure S3).

We confirmed the role of the highly conserved D146 residue, by mutating it to an aspargine, in the context of the PIN^iso1^ expression construct (PIN^D146N^), as this substitution has previously been reported to compromise the endoribonuclease activity of the PIN domain [17,19,25]. In concordance, the D146N mutation practically abolishes the activity of the PIN protein, thereby confirming its importance in Mg^2+^/Mn^2+^ co-ordination (Supplementary Figure S4). It also demonstrates that contaminating ribonucleases are not co-purified from the *E. coli* heterologous host.

To the best of our knowledge, the effect of mutating E97 in isolation has not been previously examined. Therefore, we changed the glutamic acid residue to an alanine in the context of the PIN^iso1^ expression construct (PIN^E97A^) and measured its endonucleolytic activity against a circular RNA substrate. Data presented in Supplementary Figure S5 show that the activity of PIN^E97A^ is reduced compared with the wild-type PIN^iso1^ protein. Therefore, this residue also appears to be important for the catalytic activity of the PIN domain in the context of the longer isoform.

### Modelling the effect of the shorter isoform on exosome function

**Figure 3.**
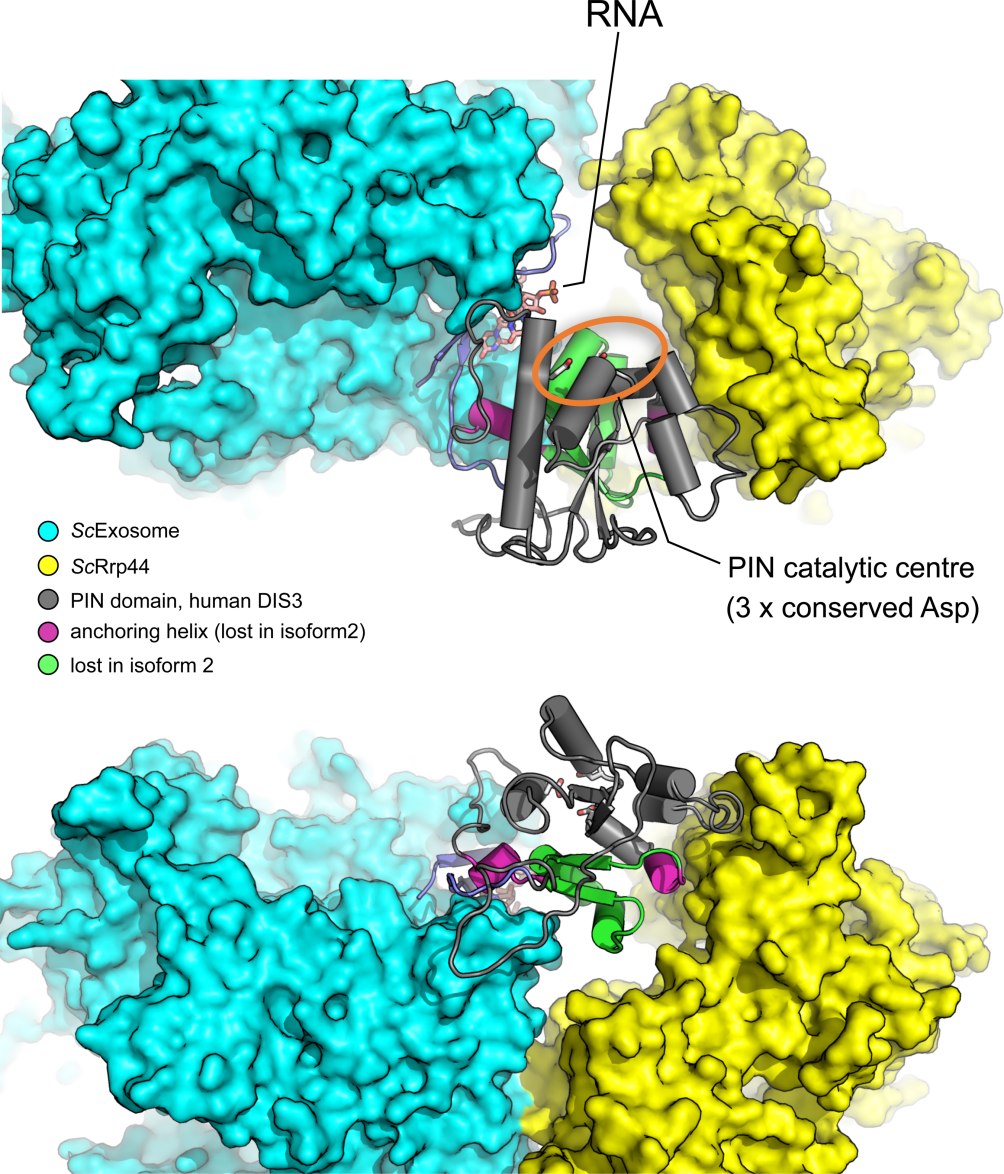
Three-dimensional model of DIS3 illustrating the possible effects of the shorter PIN domain (isoform 2) on binding of DIS3 to the exosome. The PIN domain (in grey) links the remaining part of DIS3/Rrp44 to the exosome ring complex. The catalytic site of the PIN endoribonuclease domain (three conserved aspartic acids) is marked in orange. In isoform 2, the truncated PIN domain results in loss of a loop that serves to link the PIN domain back to the extreme N-terminal part of the protein (marked in green). These key residues normally form a little loop which serves to anchor the PIN domain back to the extreme N-terminal part of the protein, facilitating the interaction with rest of the complex, as well as forming part of an RNA-interacting region. In isoform 2, the anchoring helices (purple), seen best in the lower diagram, are also missing. Therefore, isoform 2 would most probably lose the tightly constrained conformation, allowing more access to the catalytic centre of the PIN domain than would be possible in isoform 1.

Sequence-threaded models generated for both isoforms of DIS3 reveal a potential explanation for the increased catalytic activity of isoform 2. Isoform 2 deletes amino acid residues 123-FTNEHHR-129 (coloured green in Figure 3). In isoform 1, these residues form a small loop that facilitates the interaction of the PIN domain of DIS3 with the rest of the exosome complex, while also forming an integral part of an RNA-interacting region. Two additional motifs, CR3 and YxRxD, also contribute to the PIN/exosome interface [20,26]. The loss of the FTNEHHR ‘anchoring’ motif in isoform 2 is likely to result in a higher degree of conformational plasticity and flexibility for the PIN domain, generating a wider, less obstructed channel for RNA to access the endoribonuclease active centre. This could explain the greater endonucleolytic activity of isoform 2 observed in our biochemical assays.

### Isoform 1 is the principal DIS3 isoform expressed in immortalized cell lines

To experimentally validate the presence of the two annotated DIS3 isoforms, semi-quantitative reverse-transcription PCR (RT-PCR) was employed. Primers were designed to flank the variable exon 2 by annealing to exons 1 and 3 common to both isoforms (Supplementary Figure S5A), thus amplifying two fragments, each corresponding to the individual isoform transcripts. RT-PCR was performed on six cell lines of differing cancer types (Supplementary Figure S5B), predominantly AML and myeloma as well as mononuclear cells from haematological cancer patients which included six myeloma patients (Supplementary Figure S5C), four AML patients (Supplementary Figure S5D), and three CMML patients (Supplementary Figure S5E). Supplementary Figure S5 shows the amplification of two bands as expected, differing in size by exactly 100 bp. The larger fragment corresponds to isoform 1 with the full-length PIN domain and the smaller fragment to isoform 2 containing the short PIN domain. These findings illustrate that both isoform transcripts are expressed, thus corroborating the bioinformatics data. Upon initial inspection, the intensity of the isoform 1 band appears stronger than that of isoform 2 across all the cell lines and myeloma samples. In the AML samples, the intensities are more equal, while in two of the CMML patients the opposite is observed (Supplementary Figure S5E). This raises the question of whether isoform expression is tissue- or disease-specific.

**Figure 4.**
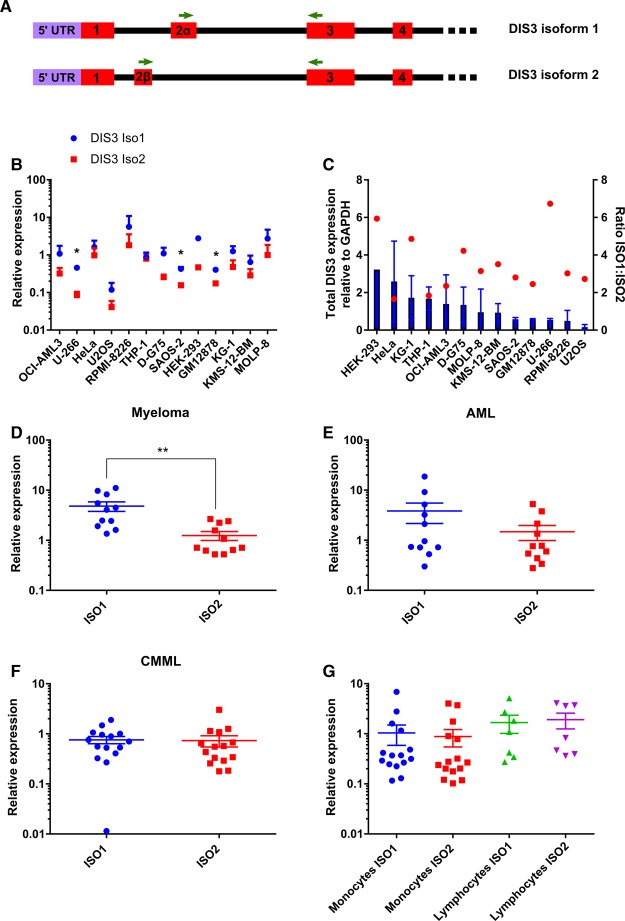
Relative expression of the two DIS3 isoform transcripts in cell lines and patient samples. (**A**) Schematic of the two DIS3 isoform transcripts with green arrows showing the position of the isoform-specific TaqMan primers. (**B**) Relative expression of the two isoforms in 13 cell lines relative to GAPDH (*n* = 3). Asterisks (in black) indicate where the relative expression is significantly different (*P* < 0.05). (**C**) Total DIS3 expression relative to GAPDH (blue bars) in 13 cell lines does not correlate with isoform ratio (red spots) (*r* = 0.14, *P* = 0.64). (**D**) In myeloma patient samples, expression levels of isoform 1 is consistently significantly higher than isoform 2 in myeloma patients (*P* = 0.0012). (**E**) In AML patient samples, isoform 1 is expressed at slightly higher levels than isoform 2. (**F**) In CMML patient samples, levels of isoforms 1 and 2 are similar. (**G**) qRT-PCR shows that monocytes and lymphocytes isolated from healthy individuals show similar levels of isoforms 1 and 2. For further details on proportions of DIS3 isoforms 1 and 2 in cell lines and patient samples, see Supplementary Figure S6.

To address this initial observation, qRT-PCR was carried out using isoform-specific TaqMan primer-probe assays (Figure 4A). As the levels of the two targets are being directly compared with each other, the amplification efficiency of the primer probes was first tested by generating a standard curve (Supplementary Figure S1). Isoform 1 is consistently more highly expressed than isoform 2 in all of the 13 cell lines tested, contributing an average of ∼70% to total DIS3 levels (Figure 4B and Supplementary Figure S6A). As isoform 1 appears to contribute to the majority of the total DIS3 transcripts expressed, an intriguing question is whether there is a correlation between the relative level of the two isoforms and the total level of DIS3 protein expressed. However, Figure 4C shows this not to be the case (*r* = 0.143, *P* = 0.640), which is perhaps not surprising given all cell lines show a similar isoform ratio.

### Higher levels of expression of isoform 1 are prevalent in myeloma cells but not in cells from AML and CMML patients

To extend the above findings on the isoform expression levels in cell lines to patient samples, qRT-PCR was employed using the same isoform-specific TaqMan primer probes as above (Figure 4A). In agreement with the semi-quantitative RT-PCR findings, all bone marrow samples from myeloma patients had a higher expression level of isoform 1 (Figure 4D and Supplementary Figure S6B). In contrast, for AML patient samples, the relative expression of isoform 1 was not significantly higher than isoform 2 with 3 out of 11 patient samples having a higher proportion of isoform 2 compared with isoform 1 (Figure 4E and Supplementary Figure S6C). For CMML patient samples, the relative expression of isoforms 1 and 2 was very similar and more of isoform 2 was expressed than isoform 1 in 5 of the 15 patient samples tested (Figure 4F and Supplementary Figure S6D).

As myeloma is a lymphoid malignancy and AML and CMML are myeloid-derived, this suggests that this expression pattern is lymphocyte-specific. To test this, peripheral blood mononuclear cells were isolated from healthy individuals before being separated into monocyte and lymphocyte fractions. Monocytes were isolated using the adherence method, and anti-CD14 staining was used to test the purity, as well as to confirm IL-6 up-regulation upon lipopolysaccharide stimulation of monocytes (data not shown). Lymphocytes remained in suspension providing an easy and effective method to isolate the two cell fractions.

**Figure 5.**
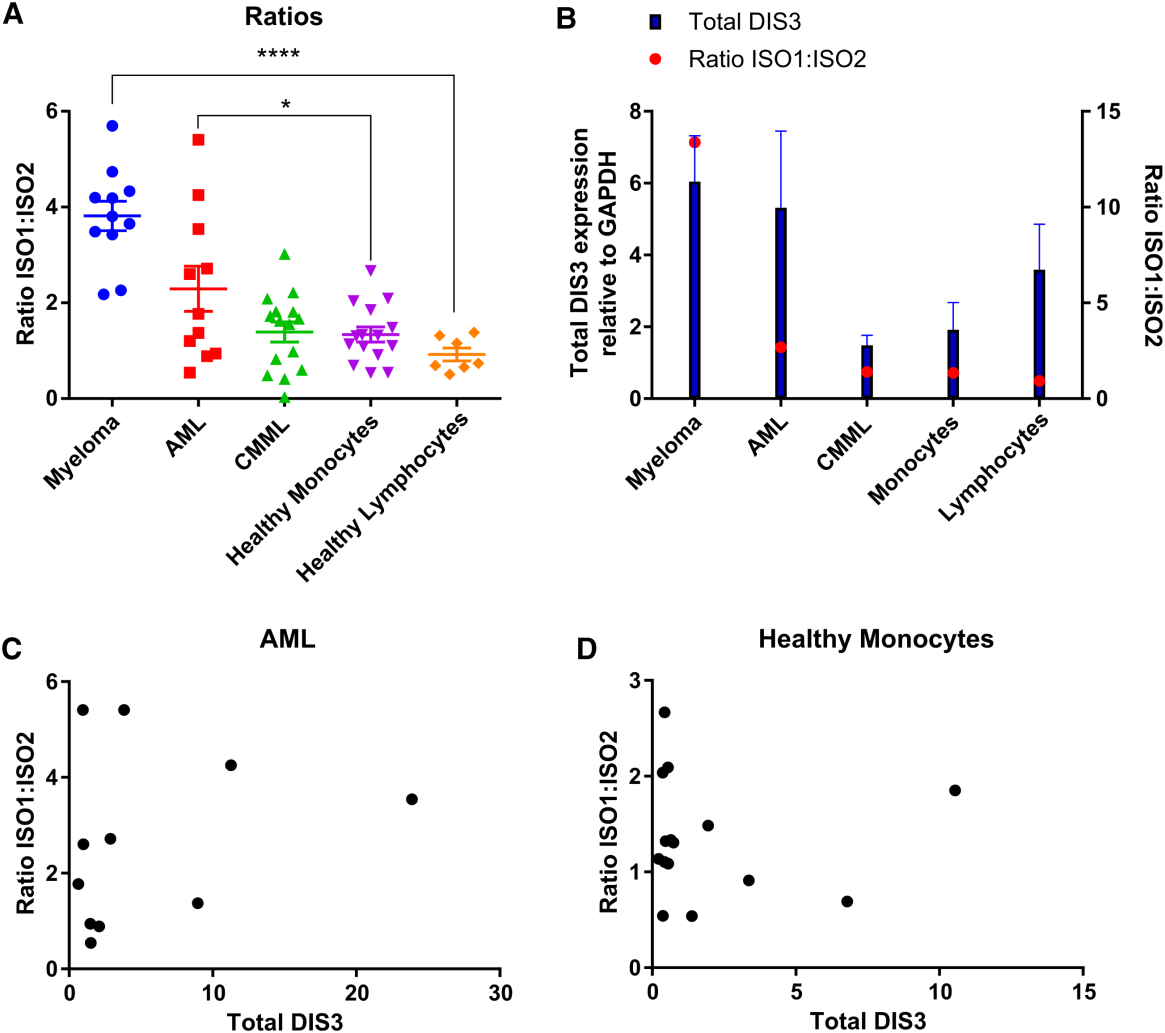
Ratio of isoform expression levels versus total DIS3 levels. (**A**) The ratio of isoform 1 to isoform 2 is significantly higher between myeloma and healthy lymphocytes (*P* < 0.0001) as well as AML and healthy monocytes (*P* = 0.04) but not between CMML and healthy monocytes (*P* = 0.84). (**B**) There is a positive correlation between the iso1 : iso2 ratio and total DIS3 levels across the disease types (*r* = 0.941, *P* = 0.017. (**C** and **D**) Within AML patients (**C**) and healthy monocytes (**D**) here is no correlation between the iso1 : iso2 ratio and total DIS3 levels.

The qRT-PCR experiments demonstrate that, in healthy monocytes, the levels of isoforms 1 and 2 are similar, with 4 out of 15 individuals having higher levels of isoform 2 than isoform 1 (Figure 4G and Supplementary Figure S6E). Nevertheless, this is not myeloid-specific as healthy lymphocytes also show the same pattern (Figure 4G and Supplementary Figure S6F). This suggests that CMML patients are more similar to healthy controls in their expression ratio of the two DIS3 isoforms compared with myeloma patients. When the ratio of isoform 1 to isoform 2 within the three diseases are compared with healthy cells, myeloma is indeed significantly different (*P* < 0.0001), whereas CMML patients are not significantly different (Figure 5). Interestingly, AML patients display a greater level of variation in isoform expression levels, but do appear to be significantly different from healthy controls (*P* = 0.04). In summary, myeloma patient samples and all cancer cell lines tested have higher levels of isoform 2, whereas CMML patient samples and samples from healthy donors have similar levels of isoforms 1 and 2.

Unlike the cell line data, there is a significant correlation between the relative level of the two isoforms and the total level of DIS3 protein expressed in these primary cells (Figure 5B, *r* = 0.941, *P* = 0.017). However, no relationship is seen when the data within individual disease types are examined (Figure 5C,D). Taken together, these data suggest that the isoform ratios observed are a result of the cell specifically regulating the expression of isoform 1 to change the stoichiometric ratio of the two isoforms, rather than as a general means of controlling total DIS3 protein levels.

### Isoform expression ratios correlate with disease severity in CMML patients

**Figure 6.**
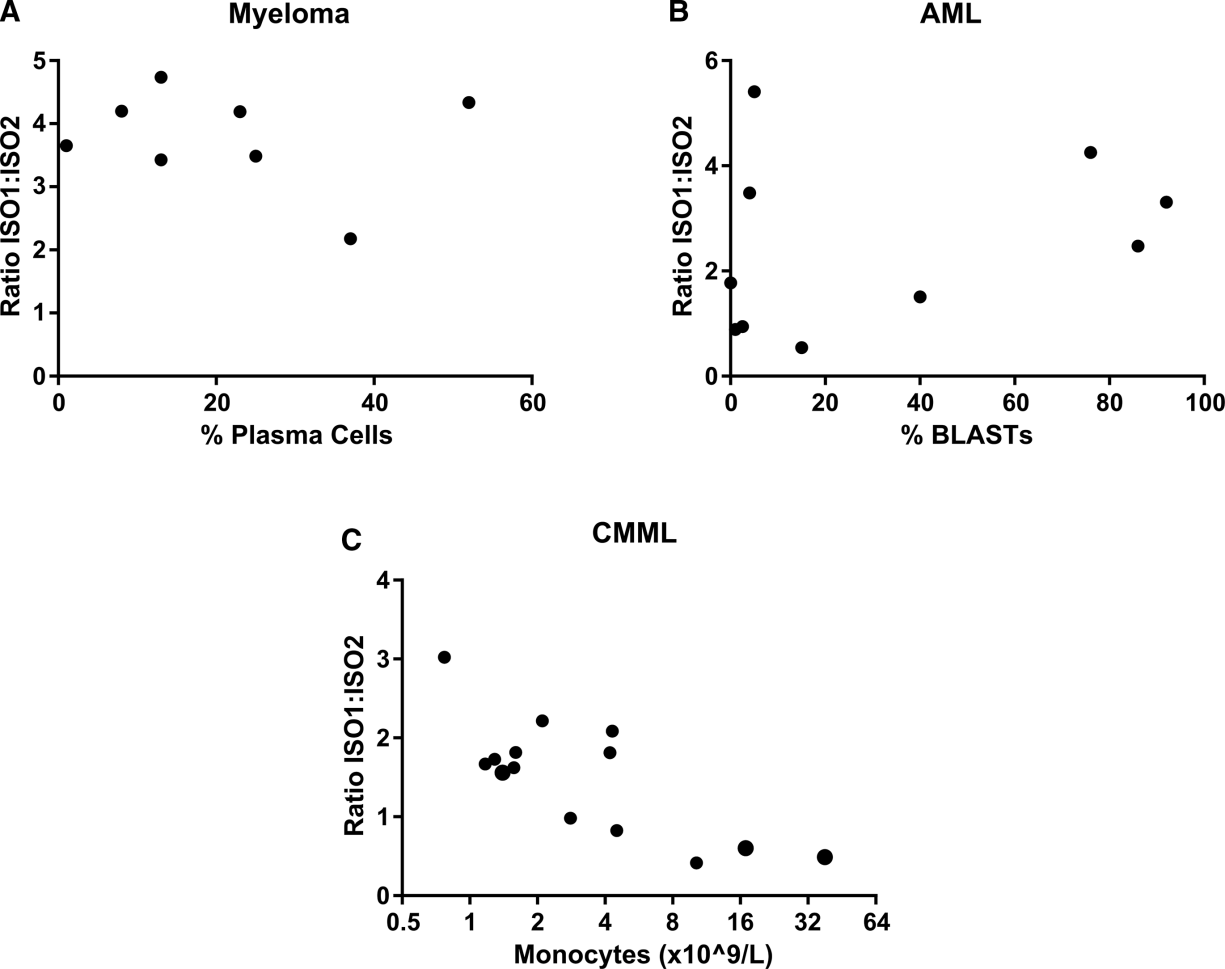
Correlation between cell count and isoform expression ratio in three haematological malignancies. The ratio of isoform 1 to isoform 2 does not correlate with plasma cell or blast count in (**A**) myeloma (*r* = −0.18, *P* = 0.67) or (**B**) AML, respectively (*r* = 0.29, *P* = 0.412). (**C**) In CMML, however, a significant negative correlation exists between the iso1 : iso2 ratio and monocyte count (*r* = −0.62, *P* = 0.018).

Given the lack of evidence for the isoform ratio being a way of modulating total DIS3 protein levels, it was of interest to find out whether there was any correlation with disease severity. This was examined using the clinical parameters of plasma cell, blast and monocyte count for myeloma, AML and CMML, respectively. Although a crude measure of disease severity alone, these cell counts are one of the parameters used to diagnose disease. Figure 6 shows that the percentage of plasma cells/blast cells is not correlated with an isoform ratio in either myeloma (*r* = −0.18, *P* = 0.67) or AML (*r* = 0.29, *P* = 0.41). However, in CMML, there was a significant negative correlation between the percentage of monocytosis and isoform expression (Figure 6C). That is, CMML patients with a higher level of isoform 2 compared with isoform 1, or a smaller ISO1 : ISO2 ratio, have a higher number of monocytes in their blood (*r* = −0.62, *P* = 0.01).

## Discussion

The human protein DIS3 is of interest due to its frequent mutation in multiple myeloma patient cells. Here, we describe the existence of two protein-coding isoforms of human DIS3 that differ in the size of their PIN domain, as a result of alternative splicing using a mutually exclusive second exon. The shorter isoform (isoform 2) deletes several amino acids from the PIN domain, which are highly conserved with yeast Dis3/Rrp44 (Figure 1). As a result, we expected it to be highly compromised in endoribonuclease activity with respect to isoform 1. However, contrary to expectation, our biochemical analyses revealed the converse to be true. We also found that DIS3 isoform 1 was expressed at higher levels in all the immortalized cell lines tested, as well as in cells from patients affected by multiple myeloma.

Our detailed biochemical and structural analyses of the two DIS3 isoforms has provided us with insights into the function of these proteins. Our modelling shows that isoform 2 lacks the 123-FTNEHHR-129 anchoring motif (coloured green in Figure 3), which in isoform 1 facilitates the interaction of the PIN domain with the rest of the exosome complex. The loss of this motif is likely to generate a wider, flexible and less obstructed channel for RNA to access the endoribonuclease active site. It is feasible that the loss of these amino acids may also allow increased direct access of RNA to the PIN domain active site, rather than threading through the centre of the exosome. This increased direct access for RNA to the PIN domain active site may be particularly important for degradation of structured nuclear RNAs such as tRNAs and ribosomal RNAs [36]. In isoform 2, the acidic residue E97 that is conserved in yeast Dis3p is missing, suggesting that it is not required for catalytic activity in this particular context. However, biochemical analyses indicate that it is required in the context of isoform 1, as the cleavage activity of PIN^iso1^ is reduced when this residue is mutated to alanine (PIN^E97A^; Supplementary Figure S4).

Our experimental data show that both of the *DIS3* transcripts are expressed in all the cancer cell lines we tested, indicating that both types of resultant protein may be required for efficient degradation of RNA. However, in all of the 13 immortalized cell lines analyzed, isoform 1 is expressed at a higher level than isoform 2 at the mRNA level, with some cell lines expressing nine times more isoform 1 than isoform 2 (HEK293). This is consistent with our results for cells from myeloma patients, which also express higher levels of isoform 1. Therefore, the isoform with the full-length PIN domain, although less active in terms of endoribonuclease activity, is often selected in highly proliferative, immortalized cells.

The importance of isoform 2, in relation to myeloma, is supported by recent data showing that the region of the PIN domain that is missing in isoform 2 is a ‘hot-spot’ for somatic mutations in humans [37]. These mutations include N87S, T93A, R108C, and a cluster of mutations at K118E, H119D, F120L and Y121S (Figures 1C and 3). Interestingly, these mutations directly abut the FTNEHHR anchoring motif. T93 is noted as a recurrent mutation and the variant Y121S is mutation in the myeloma cancer cell line OPM2. These mutations would be predicted to affect the function of isoform 1 protein and could also hinder the correct splicing of isoform 2.

The association of isoform 1 with the cancer phenotype is borne out by our results showing that it is the principal transcript in myeloma patient cells, making up ∼80% of total DIS3 protein levels in the 11 patients tested. In AML cells, overall, more patients expressed higher levels of isoform 1 than isoform 2. In contrast, monocytes and lymphocytes from healthy individuals expressed similar levels of isoforms 1 and 2. Therefore, the increase in isoform 1 in comparison with isoform 2 in myeloma cells is supportive of its role in cancer progression.

The reason for the prevalence of isoform 1 in cancer cell lines and in myeloma tumour cells is not clear. Our data suggest that reduced expression of isoform 2 may result in less degradation of target transcripts overall, particularly those that require endonucleolytic cleavage. Transcriptomic profiling of cells from myeloma patients has shown that a significant fraction of up-regulated transcripts encode proteins that are involved in RNA processing and degradation, e.g. PATL2 (protein associated with topoisomerase II homologue 2; RNA binding), DHX58 (DExH-Box Helicase 58; RNA helicase), PAN2 (PolyA-Specific Ribonuclease Subunit) and POP1 (Processing of Precursor 1; deadenylases), APOBEC3F (Apolipoprotein B mRNA Editing Enzyme Catalytic Subunit 3F; RNA editing) and RNU11 (RNA, U11 Small Nuclear; spliceosomal subunit) [37]. Therefore, mutations in DIS3 appear to be affecting post-transcriptional control pathways in the cell which presumably lead to up-regulation of specific RNAs involved in proliferation. In the case of CMML, where increased expression of isoform 2 is correlated with disease progression, it is expected that a different set of target RNAs will be up-regulated, together with mutations in other genes, resulting in alternative pathways leading to cancer phenotypes.

In conclusion, our data are consistent with human DIS3 encoding two variant forms of this protein, each of which has differences in the mode of action of their endoribonuclease activities. Aberrant expression of each of these two isoforms may contribute to the progression of haematological cancers. Further work to assess the differing effects of these two isoforms on cellular and cancer phenotypes may shed light on the progression of these cancers.

## Abbreviations

AML: acute myeloid leukaemia
CMML: chronic myelomonocytic leukaemia
DIS3: defective in sister chromatid joining
GST: glutathione *S*-transferase
PIN: PilT N-terminus
RT-PCR: reverse-transcription PCR
