# Supplementary material for: DIS3 isoforms vary in their endoribonuclease activity and are differentially expressed within haematological cancers
Source: Biochem J. 2018 Jun 29;475(12):2091–105. doi: 10.1042/BCJ20170962 (PMC6024818; doi:10.1042/BCJ20170962)
Supplement: Supplementary Tables and Figures [file BCJ-475-2091-s1.pdf]

Supplemental methods

Overexpression, purification and in vitro activity assays of the PIN truncated version of human Dis3 isoform1 and respective mutants

The vector pRSV-1, expressing the truncated version of isoform 1, composed only of the PIN domain fused to the protein purification tag, was amplified by PCR with primers that cover the entire sequence but insert alterations in their sequences to create the respective mutant versions, PIN^{E97A} and PIN^{D146N}. In each case, a silent mutation (not creating any other amino-acid change) was inserted also through each primer, to create an extra restriction site to help with the selection of the positive clones. In this case, an extra *Sca*I restriction site was inserted with E97A mutation and a *Bsm*I restriction site inserted with D146N mutation. The PCR products were circularized with T4 DNA ligase and used to transform competent DH5α strains (vectors pRSV-3 and pRSV-4 for $PIN^{\mathbf{E97A}}$ and $PIN^{\mathbf{D146N}}$, respectively). The sequence of the selected clones was confirmed by DNA sequencing.

The PIN truncated versions containing only the respective PIN domain (PIN^{iso1}, PIN^{E97A} and PIN^{D146N}) were overproduced with a Glutathione S-transferase (GST) tag in *E. coli* BL21-CodonPlus(DE3)-RIL strain containing the recombinant plasmids of interest. The proteins were overexpressed and purified by affinity chromatography (as described in the main manuscript). *In vitro* activity assays of the proteins were performed using the synthetic 30-mer oligoribonucleotide ss16-A14 as a substrate, labelled at its 5′ end with [γ-32ATP] and T4 Polynucleotide Kinase (Ambion), and circularized with T4 RNA ligase (Thermo). Protein and RNA concentrations were 250 nM and 25 nM, respectively. The experiment was performed in a buffer containing 20 mM HEPES pH 7.5, 150 mM NaCl, 3mM MnCl2 and 1 mM DTT and following the procedure described in the main manuscript.

Table S1. List of plasmids used in this work.

Plasmid	Comments	Reference
H0869	pReceiver-B03 vector expressing DIS3 ISO1 fused to a GST-tag; Amp^R	Genecopoeia
H3667	pReceiver-B03 vector expressing DIS3 ISO2 fused to a GST-tag; Amp^R	Genecopoeia
pRSV-1	pReceiver-B03 vector expressing PIN domain of DIS3 ISO1 fused to a GST-tag; Amp^R	This study
pRSV-2	pReceiver-B03 vector expressing PIN domain of DIS3 ISO2 fused to a GST-tag; Amp^R	This study
pRSV-3	pReceiver-B03 vector expressing PIN domain of DIS3 $ISO1^{\mathbf{E97A}}$ fused to a GST-tag; Amp^R	This study
pRSV-4	pReceiver-B03 vector expressing PIN domain of DIS3 $ISO1^{\mathbf{D146N}}$ fused to a GST-tag; Amp^R	This study
pGEX-4T-1	Protein expression vector with GST-tag; Amp^R	GE Healthcare

Table S2. Oligonucleotides used in this work

Name	Sequence 5'-3'	Comments
30-mer	5'-CCCGACACCAACCACU-A_{14}	RNA oligo used as substrate (circularized)
PIN FRW	5'P-CTCGAGTGCGGCCGCAACCCAGCTTTC	oligo used in the construction of pRSV-1/2
PIN REV Mut	5'P-CCACTTTCTATTT**A**ATTCCCTTCTTCAG	oligo used in the construction of pRSV-1/2
E97A FRW *Sca*I	5'P-CTACAAACAGT**A**CTTCAAG**C**AGTGAGAAATCGC	insertion of E97A mutation (vector pRSV-3)
E97 REV	5'P- CACAATTACATTCCTGATGGC	construction of pRSV-3 vector
Primer 15 (D146N) *Bsm*I	5'P-AGGGAGA**G**AATGCTAATGACAGGAAT**A**ATAGAG	insertion of D146N mutation (vector pRSV-4)
Primer 15 Rev	5'P -GTTCTTGTTCTACATAGGTTTCTCTATGGTGC	construction of pRSV-4 vector

* all the DNA primers are phosphorylated at the 5' end to enable the circularization of the vector

Table S3. List of qPCR primer/probes used within this study. N/A = no sequence information available. Anchor nucleotide = a nucleotide contained anywhere within the probe sequence. Accession numbers based on the most updated version. Information presented in accordance to MIQE guidelines.

Oligonucleotide	Accession number	Sequence (5' to 3')	Anchor nucleotide	Amplicon length
DIS31 (Forward/ Reverse)	NM_014953.4	N/A	N/A	114
DIS32 (Forward/ Reverse)	NM_001128226.2	N/A	N/A	84
GAPDH (Forward/ Reverse)	NM_002046	N/A	1087	110

Table S4. List of cell lines used in this study. The cell origins and tissue type are given in brackets).

Cell Line	Cell Type & Origin
RPMI-8226	B-lymphoblast from plasmacytoma in peripheral blood of 61 year old male with myeloma (B-lymphocyte; peripheral blood)
U-266	B-lymphoblast from plasmacytoma in peripheral blood of 53 year old male with myeloma (B-lymphocyte; peripheral blood)
KMS-12-BM	Bone marrow of a 64 year old female with myeloma (B-lymphocyte; peripheral blood)
MOLP-8	Peripheral blood of 52 year old man with myeloma (B-lymphocyte; peripheral blood)
HeLa	Cervical epithelial cell of a 31 year old female with cervical adenocarcinoma (epithelial; cervix)
SAOS-2	Bone cells from an 11 year old female with osteosarcoma (partially differentiated mesenchymal stem cells; bone)
THP-1	Monocytes from peripheral blood of a 1 year old male with acute monocytic leukaemia (monocyte: peripheral blood)
GM12878	B-lymphoblast from peripheral blood of female and transformed with Epstein bar virus
U-2OS	Bone epithelial cell from a 15 year old female with osteosarcoma (partially differentiated mesenchymal stem cells; bone)
D-G75	B-lymphoblast from pleural effusion of 10 year-old male with Burkitt's Lymphoma (B-lymphocyte; pleural effusion)
KG-1	Macrophage from bone marrow of 59 year old male with acute myelogenous leukaemia (macrophage;bone marrow)
OCI-AML3	Peripheral blood of a 57 year old male with acute myeloid leukaemia (myeloid; peripheral blood)
HEK-293	Epithelial cell from human embryonic kidney (epithelial-like; embryonic kidney)

Table S5. Summary of clinical details of human participants used in this study.

Disease	Patient	Age	Sex	Disease	Patient	Age	Sex
Myeloma	1	78	M	AML	1		
	2	69	F		2	83	M
	3	86	M		3	68	M
	4	72	M		4	74	M
	5	73	F		5	76	F
	6	71	F		6	81	M
	7	78	F		7	60	M
	8	51	F		8	46	F
	9	47	M		9	76	M
	10	65	M		10	58	M
	11	54	M		11	74	F
CMML	1	94	M				
	2	62	F				
	3	67	M				
	4	60	F				
	5	53	M				
	6	63	M				
	7	74	F				
	8	66	M				
	9	75	F				
	10	71	M				
	11	72	M				
	12	72	F				
	13	78	F				
	14	85	F				
	15	79	F				

DIS3 Isoform 1

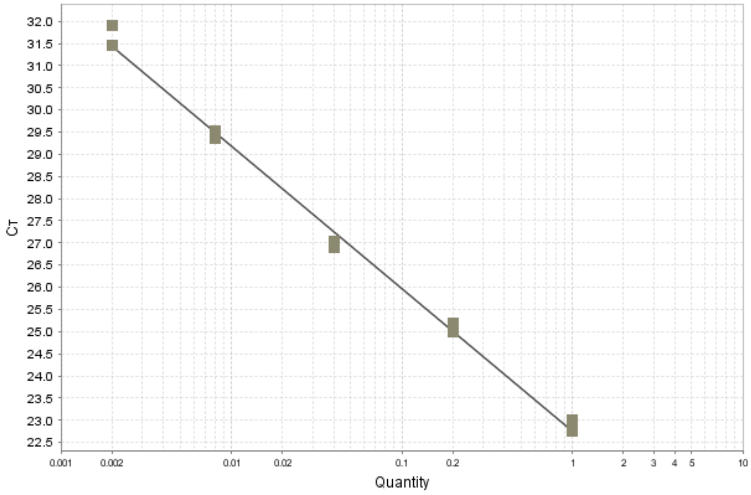

Target: dis31 Slope: -3.213 Y-Inter: 22.757 R^2: 0.996 Eff%: 104.742 Error: 0.059

DIS3 Isoform 2

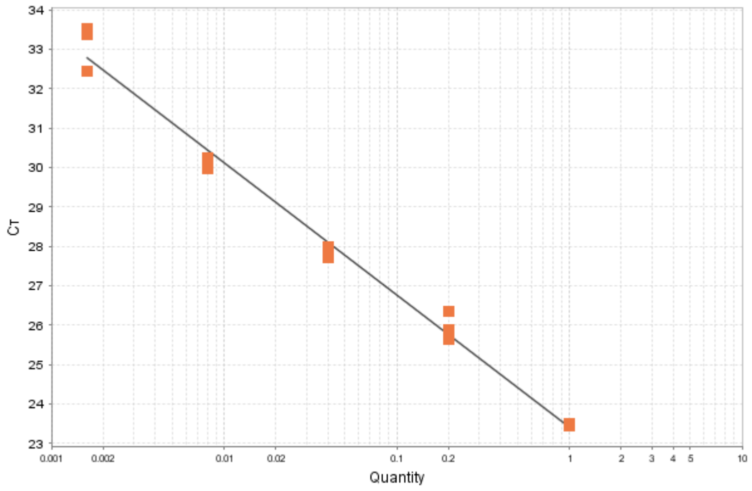

Target: dis32 Slope: -3.349 Y-Inter: 23.418 R^2: 0.987 Eff%: 98.866 Error: 0.105

Figure S1 . Standard curves and amplification efficiency of the DIS3 isoform 1 and DIS3 isoform 2 primers. (A) DIS3 isoform 1, (B) DIS3 isoform 2. Standard curves were generated using a series of cDNA dilutions (6.25, 12.5, 25, 50ng per 10ul qPCR reaction, based on RNA concentrations) and using the standard curve set up on the Life Technologies ViiA™ 7 System.

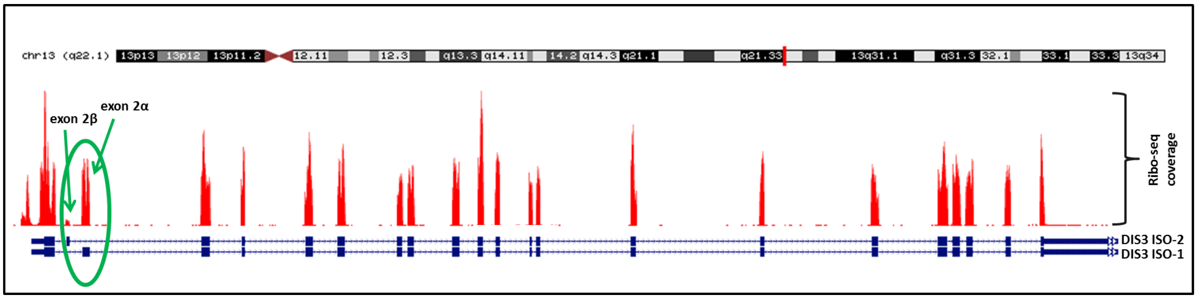

Figure S2. Visualisation of ribo-seq data on the DIS3 transcripts obtained by ribosome profiling. Although coverage is much lower on the shorter exon 2 of isoform 2 (exon 2β), ribosome binding is above background level indicating this isoform is translated. Alternative exons are labelled. Data obtained using the online tool GWIPS.

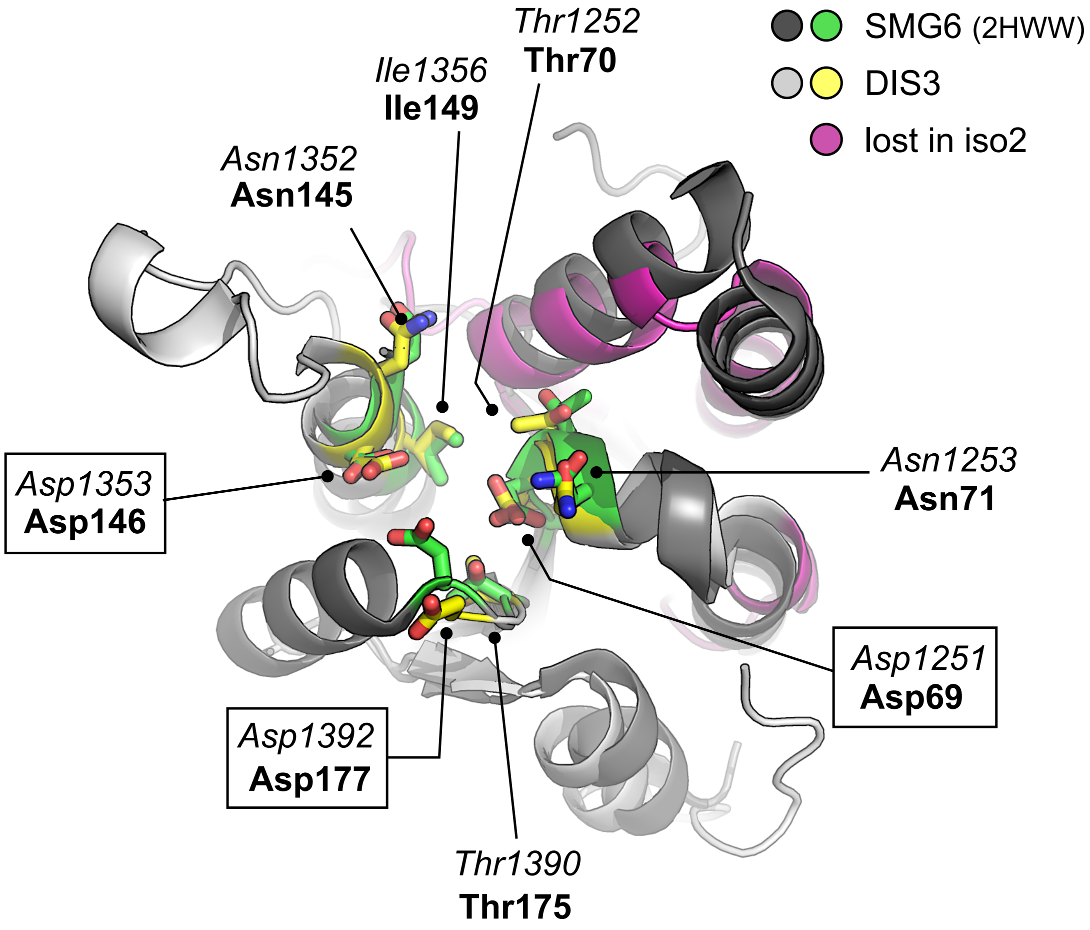

Figure S3. Diagram comparing the active sites of human DIS3 PIN domain with human SMG6. The three core catalytic residues (Asp 69, Asp 146 and Asp177) are conserved in both identity and spatial arrangements between the two proteins. The region lost in isoform 2 (purple) is unlikely to affect the catalytic activity of the PIN domain of isoform 2.

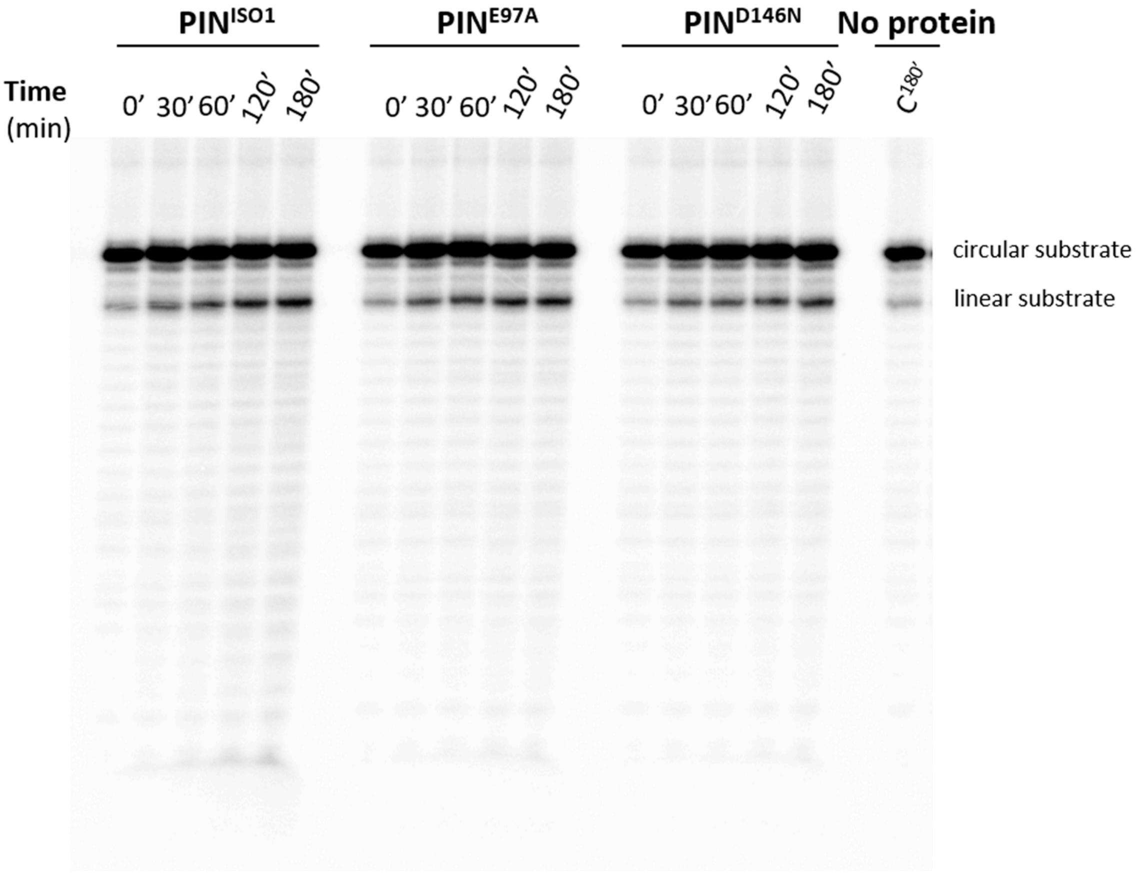

Figure S4. Effect off two single point mutations on the endoribonucleolytic activity of PIN domain on a circular RNA substrate. A 5′-labeled 30mer ssRNA circularized oligonucleotide (upper band) was incubated with a purified truncated version of human DIS3 isoforms 1 containing only the PIN domain (PINiso1) and the same protein either with a mutation in the glutamic acid residue in position 97 to an alanine (PIN^{E97A}) or a mutation in aspartic acid residue 146 to an asparagine (PIN^{D146N}). Incubation times are indicated on top of the panels. The lower band in the substrate is a linear molecule resulting from the suboptimal yield of the circularization reaction. The E97A mutation, in one of the conserved residues of PIN domain necessary for the metal ion coordination, causes a considerable reduction in activity. The activity of the protein is practically abolished by the mutation in the catalytic residue 146 (D146N), as previously reported [17,19]. For comparison of the activity of PIN^{iso1} with the respective mutants, the concentrations of protein and RNA were 250 nM and 25 nM, respectively. The experiments were performed in a buffer containing 3mM $MnCl_2$ (endonucleolytic buffer conditions). See Supplemental methods for further details.

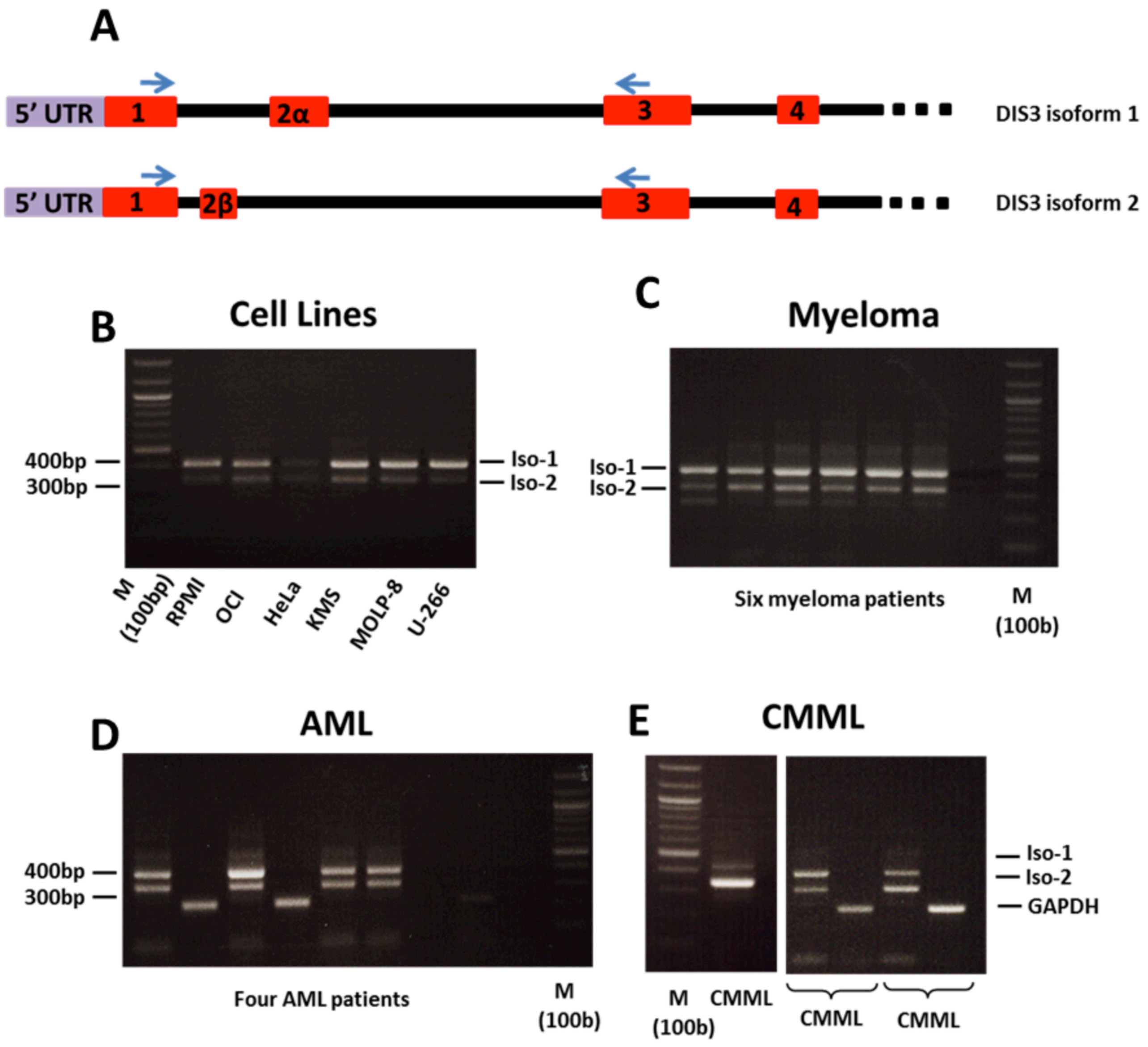

Figure S5. RT-PCR demonstrating the ubiquitous expression of the two DIS3 isoforms in different cell types. (A) Schematic of the two DIS3 isoforms with blue arrows showing the position of the primers flanking the variable exon 2 by annealing to exons 1 and 3 common to both isoforms. Two bands can be seen corresponding to isoform 1 (400bp) and isoform 2 (300bp) in cell lines (B), myeloma patients (C), AML patients (D) and CMML patients (E). GAPDH was used as a control in four of the samples.

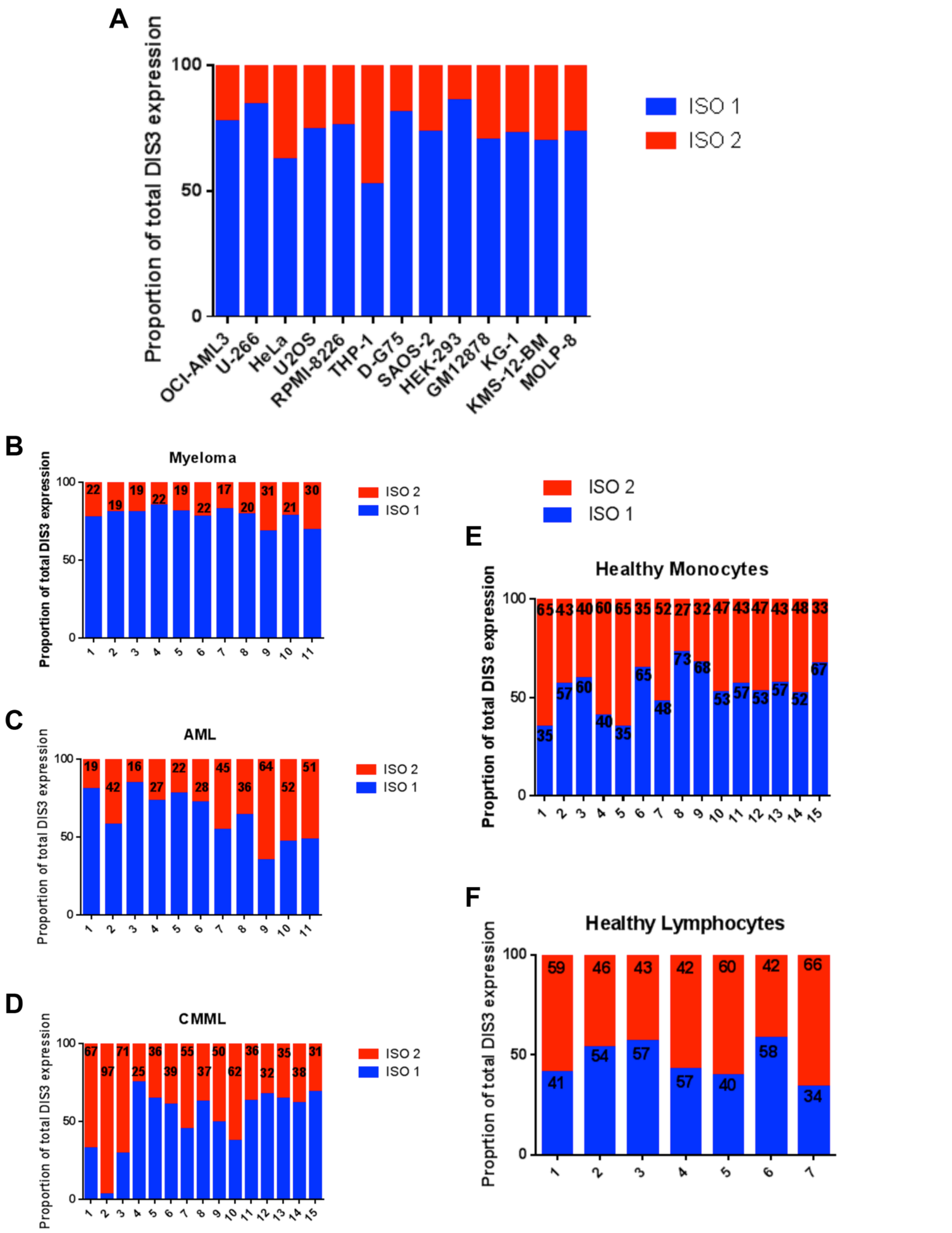

Figure S6. Expression of the two DIS3 isoforms as a proportion of total DIS3 expression (A) Expression of the two isoforms as a proportion of total DIS3 expression in a range of cell lines. (B) In myeloma patient samples the proportion of isoform 1:isoform 2 is, on average, 78:22. (C) In AML patient samples Isoform 1 is expressed at higher levels than isoform 2; the proportion of isoform 1:isoform 2 is 64:36. (D) In CMML patient samples, levels of isoform 1 and 2 are similar with an average proportion of isoform 1:isoform 2 being 53:47. (E and F) Isoform 1 and 2 contribute approximately equally to total DIS3 levels within individual patients. In healthy monocytes, the average proportion of isoform 1;isoform 2 is 65:45 whereas in healthy lymphocytes is 49:51. Proportions represent average of three biological replicates.
